# Supplementary material for: Energy aware stable path ad hoc on-demand distance vector algorithm for extending network lifetime of mobile ad hoc networks
Source: PLoS One. 2025 Apr 9;20(4):e0320897. doi: 10.1371/journal.pone.0320897 (PMC11981158; doi:10.1371/journal.pone.0320897)
Supplement: Attachment — Submitted filename: Response_to_reviewers_auresp_2.docx [file pone.0320897.s003.docx]

**REVIEWED PAPER TITLE:**

Ms. Ref. No.:  PONE-D-24-45310

**Paper Title:** **EASP-AODV: Energy Aware Stable Path Ad Hoc On-Demand Distance Vector Algorithm for Extending Network Lifetime of Mobile Ad hoc Networks**

Journal: **PLOS ONE**.

**Responses to Reviewer’s/Editor’s Comments**

**Acknowledgment from the Authors:**

We sincerely appreciate the constructive comments provided by the reviewers and the Editorial Board, which have significantly contributed to improving the quality of our manuscript. We are also grateful for the additional suggestions for further improvement and reconsideration of our work. We deeply value the time and effort the reviewers have dedicated to enhancing our manuscript. The authors’ responses to the reviewers' comments are highlighted in color in the revised manuscript.

**Additional Editor Comments:**

The paper has to be revised based on the comments provided by the reviewers, an extensive revision is required so the paper will be in very good standard.

**Reviewers' comments:**

1. Is the manuscript technically sound, and does the data support the conclusions? The manuscript must describe a technically sound piece of scientific research with data that supports the conclusions. Experiments must have been conducted rigorously, with appropriate controls, replication, and sample sizes. The conclusions must be drawn appropriately based on the data presented.

Reviewer #1: Yes

Reviewer #2: Yes

**Response from authors:** The study presents a technically sound evaluation of the EASP-AODV algorithm for extending network lifetime in MANETs.

2.      Has the statistical analysis been performed appropriately and rigorously?

**Response from authors:** The manuscript presents a rigorous statistical analysis of the EASP-AODV algorithm, ensuring significance through repeated experiments under varying conditions.

3.      Have the authors made all data underlying the findings in their manuscript fully available?

**Response from authors:** We have made all data underlying the study fully available, including simulation parameters, performance metrics, and raw experimental results.

4.      Is the manuscript presented in an intelligible fashion and written in Standard English?

**Response from authors:** The manuscript is well-structured, following a clear academic format with logically organized sections.

5.  Review Comments to the Author:

Reviewer #1:

Please use the space provided to explain your answers to the questions above. You may also include additional comments for the author, including concerns about dual publication, research ethics, or publication ethics. (Please upload your review as an attachment if it exceeds 20,000 characters)

Reviewer #1: This paper studied the "Energy-Aware Stable Path Ad Hoc On-Demand Distance Vector Algorithm for
Extending Network Lifetime of Mobile Ad hoc Networks". The proposed research manuscript, titled "Energy-Aware Stable Path Ad Hoc On-Demand Distance Vector Algorithm for Extending Network Lifetime of Mobile Ad hoc Networks," introduces the Energy Aware Stable Path Ad Hoc On-Demand Distance Vector (EASP-AODV) algorithm for enhancing the network lifetime of Mobile Ad hoc Networks (MANETs). MANETs are self-configured wireless networks where nodes act as both routers and hosts. Link breakage is a common issue due to factors like node mobility and limited energy. Traditional MANET protocols lack battery considerations, leading to energy depletion. The EASP-AODV algorithm factors in individual node energy and received signal strength to mitigate link failures and extend network lifespan. Evaluation using the NS2.35 simulation tool demonstrates improved performance metrics compared to existing protocols. Notable findings include reduced energy consumption and extended network lifetime. The authors emphasize the algorithm's efficacy in addressing energy utilization challenges in MANETs. The quality should be improved. Revision should be done for this version of the paper as follows:
*The motivation for the proposed method should be stated in the introduction.

**Response from the Author:** Mobile Ad-hoc Networks (MANETs) have gained significant interest due to the expansion of wireless devices and networks. As infrastructure-less wireless communication networks, MANETs offer dynamic mobility of nodes without geographical restrictions, extending services in infrastructure-based networks. However, challenges such as dynamic topology changes, energy constraints, bandwidth limitations, QoS issues, high end-to-end delay, and routing overhead persist. Among these, energy constraints remain an open area of research that is not fully addressed in existing MANET routing protocols. Efficient and stable paths are required for long transmission and continuous data forwarding. This research focuses on reducing link breakage caused by limited node energy and extending the network lifetime of MANETs.

* Some references are missing. At the same time, many important recent references are missing, which can support the idea of this paper, the following references can be added in the Section "References":

1- (2016). CGC: centralized genetic-based clustering protocol for wireless sensor networks using the onion approach. Telecommunication systems, 62, 657-674.‏
2- (2016). Congestion-aware routing and fuzzy-based rate controller for wireless sensor networks. Radioengineering, 25(1), 114-123.‏
3- (2023). Cluster-based routing method using mobile sinks in a wireless sensor network. International Journal of Electronics, 110(2), 360-372.‏
4- (2022). A two-level clustering based on fuzzy logic and content-based routing method in the Internet of things. Peer-to-Peer Networking and Applications, 15(4), 2142-2159.‏
5- (2022). An efficient gray system theory-based routing protocol for energy consumption management in the Internet of Things using fog and cloud computing. Computing, 104(6), 1307-1335.‏
6- (2022). An overlapping routing approach for sending data from things to the cloud inspired by fog technology in the large-scale IoT ecosystem. Wireless Networks, 28(2), 521-538.‏

**Response from the Author:** We have added and reviewed the references in the literature section

* Consider expanding on the specific methodologies used in each stage of the ECCAD model for clarity and reproducibility.

**Response from the Author:** The **ECCAD model** optimizes routing by prioritizing nodes with higher residual energy, ensuring balanced energy consumption and reducing link failures. It adjusts routing decisions dynamically based on individual node energy levels, enhancing network stability. This approach prevents early depletion of critical nodes, extending the network’s lifetime. By considering energy, the model ensures efficient resource utilization. Ultimately, it maximizes network performance and sustainability.

* How does the EASP-AODV algorithm determine individual node residual energy?

**Response from the Author:** In the EASP-AODV algorithm, individual node residual energy is monitored by tracking energy consumption during communication activities like transmission and reception. Each node updates its energy after each operation, and the residual energy is included in routing requests (RREQ). Nodes with higher residual energy are preferred for route selection to ensure efficient data transmission. This approach helps prevent early node failures due to low energy.
* What specific metrics are used to evaluate the performance of the proposed algorithm?

Response from the Author:

To evaluate the **EASP-AODV** algorithm, the following common performance metrics are typically used:

**End-to-End Delay:** End-to-end delay (EED) is the amount of time it takes for a packet to travel from the source to the destination, averaged over the total number of data packets successfully received.

**Packet Delivery Ratio:** Packet delivery ratio (PDR) is the ratio of the total number of packets successfully received by the destination to the total number of packets sent by the source.

**Normalized Routing Overhead (NRL):** Normalized routing overhead (NRL) refers to the ratio of the number of routing packets transmitted to the number of data packets successfully delivered to the destination. It provides a measure of the efficiency of the routing process relative to the actual data transmission.

**Normalized Energy Consumption (NEC):** Normalized Energy Consumption (NEC) measures network efficiency by calculating the total energy consumed by all nodes relative to the number of successfully received data packets. Since transmission and reception demand significant energy, NEC reflects the energy cost per delivered packet. Lower NEC indicates better energy efficiency and prolonged network lifespan.

**Network Lifetime:** In MANETs, network lifetime is constrained by the finite energy of nodes, making efficient energy management crucial. When a node with low energy receives a route request (RREQ), it may drop the packet, leading to higher delays and reduced packet delivery. Network lifetime is defined as the duration until nodes deplete their energy and can no longer function.

* How does the EASP-AODV algorithm reduce link breakage in MANETs?

**Response from the Author:** The **EASP-AODV algorithm** reduces link breakage in **MANETs** by prioritizing nodes with higher residual energy, ensuring stable routes. It incorporates energy-aware path selection and sets energy thresholds to avoid nodes with low energy, which reduces the likelihood of link failures. The algorithm dynamically adjusts routes based on energy levels and node stability, preventing sudden link breakages. In case of failure, EASP-AODV employs localized route recovery to quickly find alternative paths. These measures help maintain stable communication and minimize link disruptions.
* What are the key differences between EASP-AODV and traditional AODV routing protocols?

**Response from the Author:** EASP-AODV improves upon AODV by incorporating residual energy into route selection, ensuring greater stability in MANETs. While AODV chooses the shortest path based on hop count, it overlooks node energy, leading to potential link failures. EASP-AODV prioritizes energy-rich nodes, reducing disruptions and enhancing network reliability. EASP-AODV improves upon AODV by integrating residual energy into route selection, ensuring stable and energy-efficient paths in MANETs. Unlike AODV, which selects routes based solely on hop count, EASP-AODV excludes low-energy nodes, proactively maintains routes, and repairs links using energy-aware alternatives. This approach reduces link failures, extends network lifetime, and enhances reliability.
* What simulation tool was used for evaluating the proposed algorithm?

**Response from the Author:** The EASP-AODV algorithm was evaluated using NS2, a widely used discrete-event simulator for modeling network protocols, including MANETs. NS2 enabled the simulation of network topologies and performance analysis based on metrics like packet delivery ratio, end-to-end delay, and normalized energy consumption. The results compared EASP-AODV with traditional AODV, highlighting its efficiency and stability.

* How does the EASP-AODV algorithm contribute to prolonging network lifetime?

**Response from the Author:** EASP-AODV extends MANET lifetime by prioritizing energy-efficient routes, avoiding low-energy nodes, and proactively maintaining stability. It reduces link failures, minimizes control overhead, and balances energy consumption across the network. These strategies enhance reliability and ensure prolonged network performance.
* How does the EASP-AODV algorithm compare to other existing energy-aware routing protocols in MANETs?

**Response from the Author:** EASP-AODV enhances energy-aware routing by integrating stable path selection, proactive maintenance, and balanced energy distribution. By preventing link failures and minimizing control overhead, it extends network lifetime more efficiently than existing protocols. This makes it ideal for energy-constrained MANET environments.

Reviewer #2:

The present work is interesting, however, some further edits are required to enhance the readability of the work.
1. Kindly make more work in the abstract to make it more attractive and according to the work. **Response from the Author:** We have made more attractive the abstract section according to the work.

2. The literature in the introduction is very less, I can recommend literature in a broad sense to improve it further and make connection of literature with the existing work, doi: 10.1109/TVT.2018.2828651, doi: 10.1109/TSC.2024.3478730, doi: 10.1109/MNET.2024.3350025, doi: 10.1109/TVT.2023.3304707, doi: 10.1109/TMM.2024.3394681, doi: <https://doi.org/10.1287/moor.2022.1310>, doi: <https://doi.org/10.1145/3664655>, doi: 10.1109/TCOMM.2024.3409539, doi: 10.1109/TMC.2024.3455417 and some other related work in the field should be searched.

**Response from the Author:** Response from the Author: We have added and reviewed the references in the literature section

3. Equation (1-4) should be properly referenced.

Response from the Author: We have	properly referenced the equations

4. Before results and discussion some proper details of the method and related software used should be mentioned.

**Response from the Author:** The EASP-AODV algorithm enhances MANET longevity by integrating energy awareness into AODV, selecting energy-efficient routes, and dynamically adjusting paths based on real-time energy consumption. Simulated in NS-2, it demonstrated superior performance in packet delivery, energy efficiency, and network stability.

5. I recommend the authors improve the connection between sections and the obtained results should be properly linked with the method used.

Response from the Author: We have improved the connection between sections properly

6. Discussion should be enhanced in more aspects.

**Response from the Author:** This study proposed the EASP-AODV algorithm to enhance MANET lifetime by integrating energy awareness into route discovery and maintenance. Simulated in NS-2, EASP-AODV outperformed AODV in packet delivery, energy efficiency, and network stability by prioritizing energy-rich nodes and balancing load distribution. Future work will focus on optimizing scalability and refining energy models for real-world applicability.

**Comments to the Author**

1. If the authors have adequately addressed your comments raised in a previous round of review and you feel that this manuscript is now acceptable for publication, you may indicate that here to bypass the “Comments to the Author” section, enter your conflict of interest statement in the “Confidential to Editor” section, and submit your "Accept" recommendation.

Reviewer #1: All comments have been addressed

Reviewer #2: All comments have been addressed

2. Is the manuscript technically sound, and do the data support the conclusions?

The manuscript must describe a technically sound piece of scientific research with data that supports the conclusions. Experiments must have been conducted rigorously, with appropriate controls, replication, and sample sizes. The conclusions must be drawn appropriately based on the data presented.

Reviewer #1: Yes

Reviewer #2: Yes

3. Has the statistical analysis been performed appropriately and rigorously?

Reviewer #1: Yes

Reviewer #2: Yes

4. Have the authors made all data underlying the findings in their manuscript fully available?

The [PLOS Data policy](http://track.editorialmanager.com/CL0/http:%2F%2Fwww.plosone.org%2Fstatic%2Fpolicies.action%23sharing/1/010f0194d16d7efa-0e6b6b1d-3d3f-4edc-b1fa-4be0e28ed217-000000/cvVbqIhZp-BXpoQ6y-2xgMmBvkRxL_o7iJGJFIPMWFA=196) requires authors to make all data underlying the findings described in their manuscript fully available without restriction, with rare exception (please refer to the Data Availability Statement in the manuscript PDF file). The data should be provided as part of the manuscript or its supporting information, or deposited to a public repository. For example, in addition to summary statistics, the data points behind means, medians and variance measures should be available. If there are restrictions on publicly sharing data—e.g. participant privacy or use of data from a third party—those must be specified.

Reviewer #1: Yes

Reviewer #2: Yes

5. Is the manuscript presented in an intelligible fashion and written in standard English?

PLOS ONE does not copyedit accepted manuscripts, so the language in submitted articles must be clear, correct, and unambiguous. Any typographical or grammatical errors should be corrected at revision, so please note any specific errors here.

Reviewer #1: Yes

Reviewer #2: Yes

6. Review Comments to the Author

Please use the space provided to explain your answers to the questions above. You may also include additional comments for the author, including concerns about dual publication, research ethics, or publication ethics. (Please upload your review as an attachment if it exceeds 20,000 characters)

Reviewer #1: All comments have been well answered. I have no additional comments. The article is acceptable.
All comments have been well answered. I have no additional comments. The article is acceptable.

Reviewer #2: Authors revised the manuscript according to the comments. The paper is now acceptable for publication.

7. PLOS authors have the option to publish the peer review history of their article ([what does this mean?](https://track.editorialmanager.com/CL0/https:%2F%2Fjournals.plos.org%2Fplosone%2Fs%2Feditorial-and-peer-review-process%23loc-peer-review-history/1/010f0194d16d7efa-0e6b6b1d-3d3f-4edc-b1fa-4be0e28ed217-000000/vrxjAGbctuFsF1Ruluw_X_jwykD0prplREpaKjRrF1w=196)). If published, this will include your full peer review and any attached files.


If you choose “no”, your identity will remain anonymous but your review may still be made public.


**Do you want your identity to be public for this peer review?** For information about this choice, including consent withdrawal, please see our [Privacy Policy](https://track.editorialmanager.com/CL0/https:%2F%2Fwww.plos.org%2Fprivacy-policy/1/010f0194d16d7efa-0e6b6b1d-3d3f-4edc-b1fa-4be0e28ed217-000000/bDgPqwBQZJeNL6Bcd44A9Dt9SZS7Pk9b5H2DI8S8CvI=196).

Reviewer #1: No

Reviewer #2: No
