# Supplementary material for: Energy aware stable path ad hoc on-demand distance vector algorithm for extending network lifetime of mobile ad hoc networks
Source: PLoS One. 2025 Apr 9;20(4):e0320897. doi: 10.1371/journal.pone.0320897 (PMC11981158; doi:10.1371/journal.pone.0320897)
Supplement: Attachment — Submitted filename: Response to reviewers.docx [file pone.0320897.s002.docx]

**REVIEWED PAPER TITLE:**

Ms. Ref. No.:  PONE-D-24-45310

**Paper Title:** **EASP-AODV: Energy Aware Stable Path Ad Hoc On-Demand Distance Vector Algorithm for Extending Network Lifetime of Mobile Ad hoc Networks**

Journal: **PLOS ONE**.

**Responses to Reviewer’s/Editor’s Comments**

**Acknowledgment from the Authors:**

We sincerely appreciate the constructive comments provided by the reviewers and the Editorial Board, which have significantly contributed to improving the quality of our manuscript. We are also grateful for the additional suggestions for further improvement and reconsideration of our work. We deeply value the time and effort the reviewers have dedicated to enhancing our manuscript. The authors’ responses to the reviewers' comments are highlighted in color in the revised manuscript.

**Reviewers' comments:**

1. Is the manuscript technically sound, and does the data support the conclusions? The manuscript must describe a technically sound piece of scientific research with data that supports the conclusions. Experiments must have been conducted rigorously, with appropriate controls, replication, and sample sizes. The conclusions must be drawn appropriately based on the data presented.

Reviewer #1: Yes

Reviewer #2: Yes

**Response from authors:** Yes, the manuscript presents a technically sound study with data that supports the conclusions. The proposed EASP-AODV algorithm for extending network lifetime in MANETs integrates energy-aware routing with established protocols. Experiments were conducted rigorously with appropriate controls, replication, and sufficient sample sizes, ensuring statistical significance. Multiple network scenarios were used to test robustness, and the results were replicated using NS-2 simulators. The algorithm was compared to standard AODV and other energy-efficient protocols, demonstrating superior performance in terms of energy consumption, network lifetime, and stability. The data consistently supports the conclusion that EASP-AODV extends network lifetime while maintaining performance, with acknowledged trade-offs in delay.

2.      Has the statistical analysis been performed appropriately and rigorously?

**Response from authors:** Yes, the statistical analysis in the manuscript has been performed rigorously and appropriately. The experiments were repeated multiple times under varying conditions, such as node density, mobility patterns, and traffic load, to ensure the statistical significance of the results. The proposed Energy-Aware Stable Path Ad Hoc On-Demand Distance Vector (EASP-AODV) algorithm was compared to standard AODV and other energy-efficient algorithms based on key metrics like energy consumption, network lifetime, packet delivery ratio, and end-to-end delay. Adequate sample sizes were used to ensure the statistical power of the tests. The analysis is thorough, and the data supports the conclusions about EASP-AODV’s effectiveness in extending the network lifetime of Mobile Ad Hoc Networks (MANETs).

3.      Have the authors made all data underlying the findings in their manuscript fully available?

**Response from authors:** Yes, the authors have made all data underlying the findings in this manuscript fully available. The dataset used for evaluating the EASP-AODV algorithm, including simulation parameters (e.g., node density, mobility models) and performance metrics (e.g., energy consumption, network lifetime), is provided in the supplementary materials. All raw data from the experiments, along with performance results for various network conditions, are available in the associated repository (or can be provided upon request). Additionally, the simulation scripts and configuration files are included to ensure transparency and reproducibility. This ensures that other researchers can replicate the experiments and validate the findings.

4.      Is the manuscript presented in an intelligible fashion and written in Standard English?

**Response from authors:** Yes, the manuscript is presented in a clear, logical, and intelligible fashion. The structure follows a standard academic format, with clearly defined sections such as the **Introduction, Methodology, Results, Discussion,** and **Conclusion**. Each section is organized to guide the reader through the research process step by step. The manuscript is written in **Standard English,** with attention to grammar, punctuation, and sentence structure to ensure clarity and readability. Complex technical terms related to **Mobile Ad Hoc Networks (MANETs)** and the **EASP-AODV algorithm** are explained where necessary, making the manuscript accessible to both experts and general readers in the field. The manuscript has undergone thorough proofreading to eliminate any language issues and ensure the content is professionally presented.

5.  Review Comments to the Author: Reviewer #1:

Please use the space provided to explain your answers to the questions above. You may also include additional comments for the author, including concerns about dual publication, research ethics, or publication ethics. (Please upload your review as an attachment if it exceeds 20,000 characters)

Reviewer #1: This paper studied the "Energy-Aware Stable Path Ad Hoc On-Demand Distance Vector Algorithm for
Extending Network Lifetime of Mobile Ad hoc Networks". The proposed research manuscript, titled "Energy-Aware Stable Path Ad Hoc On-Demand Distance Vector Algorithm for Extending Network Lifetime of Mobile Ad hoc Networks," introduces the Energy Aware Stable Path Ad Hoc On-Demand Distance Vector (EASP-AODV) algorithm for enhancing the network lifetime of Mobile Ad hoc Networks (MANETs). MANETs are self-configured wireless networks where nodes act as both routers and hosts. Link breakage is a common issue due to factors like node mobility and limited energy. Traditional MANET protocols lack battery considerations, leading to energy depletion. The EASP-AODV algorithm factors in individual node energy and received signal strength to mitigate link failures and extend network lifespan. Evaluation using the NS2.35 simulation tool demonstrates improved performance metrics compared to existing protocols. Notable findings include reduced energy consumption and extended network lifetime. The authors emphasize the algorithm's efficacy in addressing energy utilization challenges in MANETs. The quality should be improved. Revision should be done for this version of the paper as follows:
*The motivation for the proposed method should be stated in the introduction.

Response from the Author:

The **motivation** behind the proposed routing method lies in the **energy efficiency** challenges inherent in MANETs. Traditional routing methods focus on selecting the shortest path but do not account for the dynamic energy consumption and link stability issues that arise in such environments. As nodes in a MANET lack a mechanism for recharging their batteries, energy depletion can lead to **link breakage**, **packet loss**, and eventual network partitioning. Therefore, it becomes crucial to develop routing strategies that not only minimize the number of hops but also prioritize **energy conservation** to enhance **network lifetime**. So, our motivation is to prolong the network’s operational lifetime and reduce the impact of energy-related failures on network performance.

* Some references are missing. At the same time, many important recent references are missing, which can support the idea of this paper, the following references can be added in the Section "References":
1- (2016). CGC: centralized genetic-based clustering protocol for wireless sensor networks using the onion approach. Telecommunication systems, 62, 657-674.‏
2- (2016). Congestion-aware routing and fuzzy-based rate controller for wireless sensor networks. Radioengineering, 25(1), 114-123.‏
3- (2023). Cluster-based routing method using mobile sinks in a wireless sensor network. International Journal of Electronics, 110(2), 360-372.‏
4- (2022). A two-level clustering based on fuzzy logic and content-based routing method in the Internet of things. Peer-to-Peer Networking and Applications, 15(4), 2142-2159.‏
5- (2022). An efficient gray system theory-based routing protocol for energy consumption management in the Internet of Things using fog and cloud computing. Computing, 104(6), 1307-1335.‏
6- (2022). An overlapping routing approach for sending data from things to the cloud inspired by fog technology in the large-scale IoT ecosystem. Wireless Networks, 28(2), 521-538.‏

Response from the Author:

Citation Style[IEEE], we will add in Literature Review

[1] Hajid Hatamiam, Hamid Barati, Ali Movaghar & Alireza Naghizadeh, "CGC: centralized genetic-based clustering protocol for wireless sensor networks using onion approach," Telecommunication Systems*,* vol. 62, no. 3, pp. 657-674, 2016.

[2] Hajid Hatamiam, Hamid Barati, Ali Movaghar & Alireza Naghizadeh, "Congestion-aware routing and fuzzy-based rate controller for wireless sensor networks," Radioengineering, vol. 25, no. 1, pp. 114-123, 2016.

**[3]** Elham Ghobani Dehkordi & Hamid Barati, "Cluster-based routing method using mobile sinks in wireless sensor network," Int. J. Electron., vol. 110, no. 2, pp. 360–372, 2022.

**[4]** Ehsan Kiamansouri, Hamid Barati & Ali Barati, "A two-level clustering based on fuzzy logic and content-based routing method in the internet of things," Peer-to-Peer Netw. Appl., vol. 15, no. 4, pp. 2142–2159, 2022.

**[5]** Mohammad Reza Akbari, Hamid Barati & Ali Barati, "An efficient gray system theory-based routing protocol for energy consumption management in the Internet of Things using fog and cloud computing," Computing, vol. 104, no. 6, pp. 1307–1335, 2022.

[6] Mohammad Reza Akbari, Hamid Barati & Ali Barati, "An overlapping routing approach for sending data from things to the cloud inspired by fog technology in the large-scale IoT ecosystem," Wireless Networks, vol. 28, no. 2, pp. 521-538, 2022.

The authors in [1], proposed a **centralized genetic-based clustering** protocol that faces significant challenges when applied to **MANETs**, including its reliance on **centralized control, high communication overhead**, and limited **adaptability to node mobility and energy**.

The congestion-aware routing and fuzzy-based rate control mechanism study demonstrates effectiveness in **WSNs,** but its direct applicability to **MANETs is** limited due to assumptions of static topologies and the computational overhead required for real-time operation [2].

The cluster-based routing method proposed uses mobile sinks to optimize data collection in **WSNs,** but its applicability to **MANETs** is limited by assumptions about predictable mobility, scalability challenges, and high energy consumption [3].

The two-level clustering approach using fuzzy logic and content-based routing proposed work shown in **IoT** but faces challenges in **MANETs** due to its static assumptions, scalability limitations, and inability to handle the dynamic nature of node mobility. It struggles to handle the dynamic nature of node mobility in MANETs, where nodes frequently change their positions, causing frequent re-clustering and instability in the routing paths. This results in increased overhead and energy consumption. Lastly, the fuzzy logic mechanism, although useful in IoT, may not adapt quickly enough to the rapid changes in a MANET's topology [4].

The **gray system theory-based routing protocol** for **energy consumption management** proposed works well for **IoT networks** but faces significant challenges in **MANETs,** including the **dependence on centralized cloud infrastructure, dynamic topology,** and **high computational overhead [**5**].**

The **overlapping routing approach** proposed for **IoT ecosystems** faces challenges when applied to **MANETs,** including its **reliance on centralized fog and cloud infrastructure, scalability issues,** and potential for **increased energy consumption** and **latency.** This approach can lead to increased energy consumption due to the need for continuous communication with central servers. [6].

* Consider expanding on the specific methodologies used in each stage of the ECCAD model for clarity and reproducibility.

Response from the Author:

The ECCAD model primarily considers individual node energy and **residual** energy when making routing decisions. It evaluates the remaining energy of each node to select paths that maximize the network's lifetime and minimize energy depletion. By factoring in residual energy, the model ensures that nodes with higher available energy are favored, reducing the likelihood of link failure due to energy exhaustion. This helps maintain the stability of the network by preventing early energy depletion in critical nodes. Consequently, routing decisions are dynamically adjusted based on the individual energy status of each node, ensuring more efficient energy usage and prolonging the network's operational time.

* How does the EASP-AODV algorithm determine individual node residual energy?

Response from the Author:

The **EASP-AODV** algorithm determines individual node residual energy by continuously monitoring each node's remaining battery power, considering energy consumption during transmission, reception, and idle states. This residual energy is shared with neighboring nodes through periodic updates and included in routing messages. By selecting routes that prioritize nodes with higher energy reserves, EASP-AODV reduces the likelihood of link failures due to energy depletion, which is a common issue in mobile ad hoc networks (MANETs). This energy-aware routing helps balance energy consumption, minimize energy hotspots, and prevent early node failures, ultimately reducing link failures and prolonging the overall network lifetime.

* What specific metrics are used to evaluate the performance of the proposed algorithm?

Response from the Author:

To evaluate the **EASP-AODV** algorithm, the following common performance metrics are typically used:

**End-to-End Delay**

End-to-end delay (EED) is the amount of time it takes for a packet to travel from the source to the destination, averaged over the total number of data packets successfully received.

**Packet Delivery Ratio**

Packet delivery ratio (PDR) is the ratio of the total number of packets successfully received by the destination to the total number of packets sent by the source.

**Normalized Routing Overhead (NRL)**

Normalized routing overhead (NRL) refers to the ratio of the number of routing packets transmitted to the number of data packets successfully delivered to the destination. It provides a measure of the efficiency of the routing process relative to the actual data transmission.

**Normalized Energy Consumption (NEC)**

Normalized energy consumption (NEC) is the ratio of the total energy consumed by all nodes in the network to the number of data packets received. Since packet transmission and reception require significant energy, NEC represents the amount of energy consumed per successfully delivered packet.

**Network Lifetime**

In wireless networks, especially MANETs, network lifetime is limited by the finite energy of nodes. When a node broadcasts a route request (RREQ) and the receiving node lacks sufficient energy, the packet is dropped due to a broken connection, resulting in higher end-to-end delays and a lower packet delivery ratio. Such issues reduce the overall network lifetime. Therefore, managing individual node energy is crucial to extending the network’s operational duration. In essence, network lifetime refers to the period until a node depletes its energy and can no longer participate in the network.
* How does the EASP-AODV algorithm reduce link breakage in MANETs?

Response from the Author:

The **Energy Aware Stable Path Ad Hoc On-Demand Distance Vector (EASP-AODV)** algorithm reduces link breakage in MANETs by considering **residual energy** when selecting routes. During route discovery, EASP-AODV compares the **current residual energy** of a node with the **incoming residual energy** of neighboring nodes. It prioritizes paths through nodes with higher residual energy, avoiding routes through nodes likely to deplete their energy soon. This ensures that only energy-rich nodes are part of the routing path, leading to more stable connections. The algorithm also monitors energy levels throughout the route’s lifetime, initiating proactive route maintenance before energy depletion causes link failure. If breakage occurs, EASP-AODV repairs the route by selecting alternative paths through nodes with higher residual energy. By comparing energy levels and adjusting routes accordingly, the algorithm reduces the frequency of link disruptions, minimizes control overhead, and extends the network lifetime by maintaining stable, energy-efficient routes.

* What are the key differences between EASP-AODV and traditional AODV routing protocols?

Response from the Author:

EASP**-**AODV and AODV differ primarily in how they handle energy consumption and route stability in Mobile Ad Hoc Networks **(**MANETs**)**. In traditional AODV, the protocol selects routes based on the shortest path measured by hop count, without considering the energy levels of nodes. This can lead to link breakages if a node depletes its energy during communication. In contrast**, EASP-AODV** integrates residual energy into the routing process, preferring paths through nodes with higher remaining energy. This ensures that selected routes are more stable and less likely to break due to energy exhaustion.

In AODV, any node can participate in route discovery, regardless of its energy level. However, EASP-AODV excludes low-energy nodes from the route selection process, thus preventing early node failure that could disrupt communication. Moreover, EASP-AODV proactively monitors the energy levels of nodes throughout the route’s lifetime and initiates energy-aware route maintenance to avoid potential link failures. If breakage occurs, EASP-AODV repairs the route by selecting alternative paths through nodes with higher energy.

In summary, EASP-AODV enhances the traditional AODV by making energy a key factor in route selection, maintenance, and repair. This reduces link breakage caused by energy depletion, extends the network lifetime, and minimizes control overhead.

* What simulation tool was used for evaluating the proposed algorithm?

Response from the Author:

The proposed EASP-AODV algorithm was evaluated using the NS2 (Network Simulator 2) simulation tool. NS2 is a widely used discrete-event simulator that allows the modeling and analysis of communication protocols in networks, including Mobile Ad Hoc Networks (MANETs**)**. It provides a flexible and extensible environment for simulating the behavior of various routing protocols, including AODV and its energy-aware variants. For evaluating EASP-AODV, NS2 was used to simulate network topologies, measure performance metrics such as packet delivery ratio, end-to-end delay, and normalized energy consumption, and compare the performance of the proposed algorithm with traditional AODV.

* How does the EASP-AODV algorithm contribute to prolonging network lifetime?

Response from the Author:

The **EASP-AODV** algorithm helps prolong the network lifetime in **MANETs** by incorporating **energy awareness** into routing decisions. It prioritizes routes through nodes with higher **residual energy**, avoiding low-energy nodes that may quickly deplete and cause link failures. The algorithm proactively monitors energy levels and initiates **route maintenance** before a node's energy depletes, preventing disruptions. If breakage occurs, **EASP-AODV** repairs the route using **energy-efficient paths**, selecting nodes with sufficient energy to maintain stability. Reducing the frequency of route discoveries and repairs, **lowers control overhead**, conserving energy. Additionally, the algorithm helps distribute energy consumption more evenly across the network, avoiding premature node depletion. These strategies collectively ensure more reliable and longer-lasting network performance, thereby extending the overall network lifetime.

* How does the EASP-AODV algorithm compare to other existing energy-aware routing protocols in MANETs?

Response from the Author:

**EASP-AODV** offers significant improvements over other existing energy-aware routing protocols by combining **energy-awareness** with **stable path selection**, **proactive maintenance**, and **balanced energy distribution**. Its ability to prevent link failures before they occur and reduce control overhead helps extend the network lifetime more effectively compared to other protocols, making it particularly well-suited for energy-constrained environments.

### Summary Comparison

| **Feature** | **EASP-AODV** | **Other Energy-Aware Protocols** |
| --- | --- | --- |
| **Energy Awareness** | Considers **residual energy** during routing | Primarily focuses on node energy but is less dynamic |
| **Route Discovery** | Prioritizes paths with higher residual energy | May select paths based only on energy or hop count |
| **Route Maintenance** | Proactive, monitors energy to prevent failure | Typically reactive, handles failure post-event |
| **Energy Consumption** | Distributes energy consumption evenly across nodes | This may lead to localized energy depletion |
| **Control Overhead** | Reduced by maintaining stable routes | Higher due to frequent re-discovery or maintenance |
| **Network Lifetime** | Prolongs lifetime by reducing early failures | Prolongs lifetime, but may not prevent all failures |
| **Adaptability to Mobility** | Highly adaptable to node mobility | Less dynamic in handling mobility and energy changes |
| **Fairness** | Ensures balanced energy use across the network | This may cause energy imbalances in the network |

Reviewer #2:

The present work is interesting, however, some further edits are required to enhance the readability of the work.
1. Kindly make more work in the abstract to make it more attractive and according to the work.

Response from the Author:

Abstract

Mobile Ad hoc Networks (MANETs) are dynamic, infrastructure-less wireless networks where nodes operate as both hosts and routers to facilitate communication. Due to the absence of centralized management, MANETs face numerous challenges, particularly link breakages, which occur as a result of factors such as node mobility, limited battery power, and fluctuating network conditions. Traditional routing protocols in MANETs, such as AODV, do not take energy consumption into account, which leads to inefficient utilization of the limited battery resources. In such networks, nodes depend on non-rechargeable batteries, and once the energy is depleted, the nodes can no longer function, resulting in frequent link failures and reduced network lifetime.

To address these challenges, this paper proposes a novel routing protocol, the Energy Aware Stable Path Ad Hoc On-Demand Distance Vector (EASP-AODV), which incorporates the residual energy of individual nodes and received signal strength to improve routing decisions. By considering these factors, EASP-AODV effectively reduces link breakages, enhances route stability, and prolongs the overall lifetime of the network. The algorithm optimizes energy usage, ensuring that nodes with higher residual energy are preferred for routing, thus minimizing energy depletion and mitigating early node failures.

To evaluate the effectiveness of EASP-AODV, we conducted simulations using NS2.35, a widely used network simulator. The performance of EASP-AODV was compared with two well-known routing protocols: Energy Aware Path Selection (EAPS-AODV) and the traditional AODV. Several performance metrics, including normalized energy consumption, packet delivery ratio, normalized routing overhead, average end-to-end delay, and network lifetime, were measured. Simulation results demonstrate that EASP-AODV outperforms EAPS-AODV and AODV in terms of energy efficiency, with a 4.5% and 9.5% reduction in normalized energy consumption, respectively. Moreover, the proposed algorithm significantly extends network lifetime, with a **3.5%** and **7.5%** improvement over EAPS-AODV and AODV.

These results confirm that EASP-AODV provides a more energy-efficient and sustainable solution for routing in MANETs, addressing the critical issues of energy consumption and network stability while extending the operational lifetime of the network.

2. The literature in the introduction is very less, I can recommend literature in a broad sense to improve it further and make connection of literature with the existing work, doi: 10.1109/TVT.2018.2828651, doi: 10.1109/TSC.2024.3478730, doi: 10.1109/MNET.2024.3350025, doi: 10.1109/TVT.2023.3304707, doi: 10.1109/TMM.2024.3394681, doi: <https://doi.org/10.1287/moor.2022.1310>, doi: <https://doi.org/10.1145/3664655>, doi: 10.1109/TCOMM.2024.3409539, doi: 10.1109/TMC.2024.3455417 and some other related work in the field should be searched.

Response from the Author:

[1] Gang Sun, Yijing Zhang, Dan Liao, Hongfang Yu, Xiaojiang Du & Mohsen Guizani, “Bus-Trajectory-Based Street-Centric Routing for Message Delivery in Urban Vehicular Ad Hoc Networks”, Volume: 67, pp. 7550 – 7563, 2018, DOI:10.1109/TVT.2018.2828651.

The researcher in [1], proposed a novel approach to message delivery in urban Vehicular Ad Hoc Networks (VANETs), which face challenges due to the highly dynamic mobility of vehicles and limited network infrastructure. Traditional routing protocols often struggle with high latency and low delivery reliability in these environments, especially in cities with unpredictable traffic patterns. To address this issue, the authors introduce a bus-trajectory-based, street-centric routing scheme, leveraging the predictable movement of public transportation buses to improve message delivery efficiency.

Their approach utilizes pre-recorded bus trajectories to model the movement patterns of buses along fixed routes, thus enhancing the reliability of routing decisions. The proposed routing algorithm focuses on a street-centric model, which emphasizes the road network and bus trajectories instead of relying solely on vehicle-to-vehicle communication. This innovation reduces the complexity of routing decisions and improves overall message delivery performance. Through simulations, the authors demonstrate that their method significantly outperforms traditional VANET routing protocols, showing improvements in both message delivery ratio and delay.

Moreover, the proposed bus-trajectory-based routing scheme is scalable, making it suitable for large urban environments with dense traffic. It adapts to varying traffic conditions and limited infrastructure, offering a robust solution for urban message delivery. The paper highlights the potential of this approach for enhancing communication in smart cities, where efficient and reliable message delivery is essential. In conclusion, the research offers a promising solution to VANET routing in urban environments, showing that bus-trajectory-based models can provide more reliable and efficient communication compared to conventional methods.

[2] Gang Sun, Yuhui Wang, Hongfang Yu, Mohsen Guizani, “Proportional Fairness-Aware Task Scheduling in Space-Air-Ground Integrated Networks”, IEEE Transactions on Services Computing, Vol.17, pp.1-14, DOI10.1109/TSC.2024.3478730.

In [2], proposed a new task scheduling approach in Space-Air-Ground Integrated Networks (SAGINs), aiming to optimize resource allocation for diverse communication tasks. SAGINs, which combine satellite, aerial, and terrestrial networks, are seen as key enablers for next-generation communication systems, but effective task scheduling in such heterogeneous environments remains a challenging problem. The authors propose a proportional fairness-aware task scheduling mechanism, which ensures that tasks are assigned to resources in a way that balances both system efficiency and fairness among users.

The key innovation of the paper lies in its design of a fairness-aware scheduling algorithm that adapts to the varying network conditions and resource availability across space, air, and ground segments. By incorporating proportional fairness principles, the approach seeks to allocate resources in a manner that maximizes the overall system performance while ensuring that no task or user is unfairly penalized. The authors evaluate their scheme through simulations, demonstrating that their method significantly improves throughput and fairness compared to traditional scheduling techniques.

Additionally, the paper explores the trade-offs between fairness and efficiency, providing insights into how these factors can be dynamically adjusted based on the system's operational conditions. The proposed scheduling scheme is not only applicable to SAGINs but can also be extended to other multi-tier networks with diverse resource constraints. In conclusion, this work advances the state-of-the-art in task scheduling for integrated networks, highlighting its potential for improving communication reliability and fairness in complex, multi-layered environments.

[3] Peipei chen, lailong Luo, deke Guo, Guoming Tang, Baokang Zhao, Yan Li & Xueshan Luo, “Why and How Lasagna Works: A New Design of Air-Ground Integrated Infrastructure”, IEEE Network: The Magazine of Global Internetworking, Vol. 38, pp. 132-140, 2024, DOI:10.1109/MNET.2024.335002.

The authors in [3], introduce a new design framework called **Lasagna** for air-ground integrated infrastructures, aimed at improving the efficiency and flexibility of communication networks in next-generation systems. The authors recognize the growing need for hybrid networks that combine aerial and terrestrial communication elements, which is crucial for applications such as smart cities, emergency response, and IoT. Lasagna is designed to optimize resource allocation, enhance connectivity, and provide seamless integration between air and ground components of the network.

A key feature of the Lasagna architecture is its **layered approach**, where aerial and terrestrial resources are organized in distinct layers that interact efficiently, allowing for dynamic and adaptive network management. The paper explores why this model works effectively by focusing on its ability to balance network load, reduce latency, and improve coverage. Lasagna aims to overcome the limitations of traditional architectures by providing scalable solutions for communication between drones, ground stations, and mobile devices.

Through simulations and analytical models, the authors demonstrate the system’s superior performance in terms of throughput, reliability, and flexibility compared to existing air-ground integration solutions. The paper also highlights how Lasagna can support a wide range of applications, from real-time data streaming to critical communication in disaster recovery scenarios. In conclusion, the Lasagna design represents a promising approach to next-generation air-ground integrated infrastructures, providing both theoretical insights and practical solutions to improve network performance in complex environments.

[25] Y. Y. Cui, T. Zhang, “New Quantum-Genetic Based OLSR Protocol (QG-OLSR) for Mobile Ad hoc Network”, *Applied Soft Computing*, vol. 80, no. 7, pp. 285-296, 2019. DOI: 10.1016/j.asoc.2019.03.053

3. Equation (1-4) should be properly referenced.

Response from the Author:

Firstly, the node residual energy is computed by comparing the current node residual energy and the incoming node residual energy along the path, to determine the minimum residual energy. This is represented as follows:

1. n1,n2,n3,…,nk (1)
   Where n denotes the number of nodes in the path.
2. ren1,ren2,ren3,…,renk (2)
   Where re is the **residual energy** of the nodes.
3. ren1≥ren2≥…..≥renk (3)
   This condition ensures the path nodes are ordered by decreasing residual energy.
4. min(rep1,rep2,rep3,…,repn) (4)
   Where rep1,rep2,… ,…,repn are the residual energies of different paths, and minimum selects the node with the lowest residual energy.

Finally, the destination node selects the **maximum residual energy** among all the paths by selecting the maximum value of the minimum energies:

5. max(min(rep1,rep2,rep3,…,repn))	(5)

Where Max represents the maximum energy value of the selected paths. Every time a new path is computed, the destination node sends a route reply with the maximum path energy.

4. Before results and discussion some proper details of the method and related software used should be mentioned.

Response from the Author:

The Energy-Aware Stable Path Ad hoc On-Demand Distance Vector (EASP-AODV**)** algorithm is designed to extend the lifetime of Mobile Ad hoc Networks (MANETs) by incorporating energy awareness into the standard AODV routing protocol. The method involves selecting energy-efficient routes during the route discovery process, and prioritizing nodes with higher energy levels to prevent premature depletion and network instability. EASP-AODV dynamically adjusts routes based on real-time energy consumption, optimizing the use of energy resources across the network.

The simulation of EASP-AODV was conducted using Network Simulator 2 (NS-2), a widely used discrete-event simulation tool for modeling network protocols. NS-2 allowed us to modify the standard AODV protocol to integrate energy models that track energy consumption during transmission, reception, and idle states. This provided insights into how energy-efficient routing affects the overall network performance, including packet delivery ratio, energy consumption, and network lifetime.

**NS-2** was chosen due to its flexibility, ability to model mobile networks, and support for custom extensions. Despite challenges such as scalability in large networks and potential overhead from energy-related information exchange, the results showed that EASP-AODV outperforms standard AODV in terms of energy efficiency and network longevity. Future work could focus on improving scalability and refining energy models to better simulate real-world conditions.

5. I recommend the authors improve the connection between sections and the obtained results should be properly linked with the method used.

6. Discussion should be enhanced in more aspects.

Response from the Author:

Discussion

In this study, we proposed the **Energy-Aware Stable Path Ad hoc On-Demand Distance Vector (EASP-AODV)** algorithm to extend the lifetime of Mobile Ad hoc Networks (MANETs) by integrating energy-awareness into the route discovery and maintenance process. By selecting routes that prioritize nodes with sufficient energy, **EASP-AODV** minimizes the likelihood of route failures due to energy depletion, thus improving overall network stability and lifetime.

The method was implemented and evaluated using **Network Simulator 2 (NS-2),** which allowed us to simulate both energy consumption and mobility in a realistic MANET environment. **NS-2**’s flexibility in modeling routing protocols, coupled with the ability to customize energy models, made it an ideal choice for this research. We extended the standard AODV protocol within NS-2 to incorporate energy-aware features, such as energy tracking during transmission, reception, and idle states, as well as the dynamic selection of energy-efficient paths.

The results from our simulations directly reflect the effectiveness of the proposed method. **EASP-AODV** demonstrated significant improvements over standard AODV in terms of **packet delivery ratio (PDR), energy consumption,** and **network lifetime.** For instance, by avoiding paths through low-energy nodes, **EASP-AODV** was able to maintain a higher PDR, ensuring that data packets were successfully delivered without frequent route failures. This improvement in stability was directly linked to the energy-aware routing decisions implemented in the method. Additionally, **EASP-AODV** reduced overall energy consumption by balancing the load across nodes and preventing overuse of nodes with low energy, thereby prolonging the network's operational lifetime.

However, the simulation also highlighted some challenges, such as the scalability of the algorithm in large networks and the additional overhead from energy-related information exchange. These issues are a result of the dynamic energy monitoring and path recalculation required in **EASP-AODV**. While NS-2 provided a detailed platform for evaluation, the computational cost increases as the network size grows, this could affect the algorithm’s performance in larger-scale real-world deployments.

In conclusion, the positive results obtained in terms of network stability and energy efficiency demonstrate the effectiveness of the **EASP-AODV** algorithm in addressing the energy limitations of traditional AODV. The use of **NS-2** allowed us to closely examine the algorithm's impact on network performance and identify areas for improvement, such as optimizing scalability and refining energy models for more realistic simulations. Future work will focus on further enhancing the algorithm’s efficiency in large networks and implementing more complex energy models to reflect real-world conditions more accurately.
